# Roles of Hcp family proteins in the pathogenesis of the porcine extraintestinal pathogenic *Escherichia coli* type VI secretion system

**DOI:** 10.1038/srep26816

**Published:** 2016-05-27

**Authors:** Ying Peng, Xiangru Wang, Jin Shou, Bingbing Zong, Yanyan Zhang, Jia Tan, Jing Chen, Linlin Hu, Yongwei Zhu, Huanchun Chen, Chen Tan

**Affiliations:** 1 State Key Laboratory of Agricultural Microbiology, College of Veterinary Medicine, Huazhong Agricultural University, Wuhan, Hubei, 430070, China; 2 The Cooperative Innovation Center for Sustainable Pig Production, Key Laboratory of development of veterinary diagnostic products of Ministry of Agriculture, Huazhong Agricultural University, Wuhan, Hubei, 430070, China

## Abstract

Hcp (hemolysin-coregulated protein) is considered a vital component of the functional T6SS (Type VI Secretion System), which is a newly discovered secretion system. Our laboratory has previously sequenced the whole genome of porcine extraintestinal pathogenic *E. coli* (ExPEC) strain PCN033, and identified an integrated T6SS encoding three different *hcp* family genes. In this study, we first identified a functional T6SS in porcine ExPEC strain PCN033, and demonstrated that the Hcp family proteins were involved in bacterial competition and the interactions with other cells. Interestingly, the three Hcp proteins had different functions. Hcp2 functioned predominantly in bacterial competition; all three proteins were involved in the colonization of mice; and Hcp1 and Hcp3 were predominantly contributed to bacterial-eukaryotic cell interactions. We showed an active T6SS in porcine ExPEC strain PCN033, and the Hcp family proteins had different functions in their interaction with other bacteria or host cells.

The type VI secretion system (T6SS) is a novel secretion system first identified in *Vibrio cholerae*^1^. T6SSs occur widely in the Gram-negative Proteobacteria, and are thought to be composed of 15–20 highly conserved core proteins, which form the apparatus^2^. T6SSs are frequently involved in pathogenicity of many bacteria, including *Vibrio cholerae*^3^, *Aeromonas hydrophila*^4^, *Pseudomonas aeruginosa*^5^ and *Burkholderia pseudomallei*^6^. Increasing numbers of effectors have recently been shown to contribute to bacterial competition^7^,^8^.

T6SSs have been identified in many of the pathogenic *E. coli* strains that have been sequenced, and interestingly, more than one integrated T6SS or multiple copies of *hcp* and *vgrG* genes are found in single T6SS clusters in many bacteria^4^,^6^. T6SS can influence type 1 fimbria expression and pathogenicity of avian pathogenic *E. coli* (APEC) strain SEPT362, and IcmF and Hcp are involved in the bacterial interaction with endothelial cells, reducing their adherence and inducing actin rearrangements^9^,^10^. Another APEC K1 strain, TW-XM, encodes two T6SSs in its genome^11^. T6SS2 exclusively and significantly influences its interaction with the mouse brain microvascular endothelial cell (BMEC) line, bEnd.3. T6SS1 is multifunctional, and is involved in bacterial-eukaryotic cell interactions in many cell models, in biofilm formation and in bacterial competition^11^. Neonatal meningitis *E. coli* (NMEC) K1 strain RS218 has been shown to encode two Hcp proteins of an integrated T6SS, which are involved in different stage when the bacterium interacts with human brain microvascular endothelial cells (HBMECs)^12^. Two independent T6SSs are identified in enteroaggregative *E. coli* (EAEC) 042 13. T6SS Sci-1 is required for biofilm formation and T6SS Sci-2 confers a growth advantage over *E. coli* K12 strain W3110 13.

Hemolysin-coregulated protein (Hcp) and valine-glycine repeat protein G (VgrG) are considered the hallmarks of T6SS, and because they are secreted into the extracellular environment, they can be detected in the supernatants of all bacteria with functional T6SSs^5^,^12^,^14^. The *hcp* gene is originally found in *V. cholerae*, and named according to its coordinated regulation with hemolysin^15^. Individual Hcp protein is thought to form homohexameric rings with an inner diameter of 40 Å, which act as the structural secretion components of the T6SS, allowing effectors to pass through their centres^5^. Their crystal structure and transmission electron microscopic images have shown that these hexameric rings stack on top of one another, forming a nanotube, the inner surface of which binds effector molecules^3^,^16^. Hcp is also considered to be an important chaperone for T6SS effectors, preventing their degradation, and is secreted together with them^17^. Besides its structural function in the T6SS, Hcp is thought to be a secretory protein, with multiple functions in different bacteria. It has been shown to target macrophages and inhibit phagocytosis, thus modulating the innate immunity of *A. hydrophila* SSU^4^. NMEC RS218 encodes a T6SS containing two Hcp proteins that play different roles in the bacterial interaction with HBMECs^12^. Hcp1 contributes to the rearrangement of actin, apoptosis and the release of cytokines as effectors secreted into the extracellular environment^12^. However, Hcp2 localizes to the cytoplasm and is involved in bacterial adherence and invasion in a cell model^12^. The *B. pseudomallei* K96243 genome encodes six T6SSs, but the gene cluster of only one T6SS has been identified as functional because the *hcp1* mutant shows significantly reduced pathogenesis and weak cytotoxicity against RAW264.7 cells^6^. Recently, T6SS has been considered useful in bacterial competition. T6SS1 is involved in the growth competition between *E. coli* K12 strain W3110 and EAEC 042, and the *H*cp family proteins contribute to the bacterial competition between *P. fluorescens* and *Dickeya dadantii*^13^,^18^,^19^.

Using gene sequencing, we found an integrated T6SS in the chromosome of porcine ExPEC strain PCN033, with three copies of *hcp*. Two of the *hcp* genes are located in the T6SS cluster, whereas the third is at quite a distance from it. To define the functions of each Hcp protein of porcine ExPEC strain PCN033, this study constructed markerless deletion mutants of each *hcp* gene and a triple mutant, and monitored their capacity to compete with *E. coli* K12 strain W3110. We also examined the role of the Hcp proteins in bacterial adherence, invasion and intracellular survival. Our findings showed that the T6SS in porcine ExPEC strain PCN033 had multiple functions and that the three Hcp proteins functioned in distinct ways in bacterial competition and the bacterial interactions with eukaryotic cells.

## Results

### Integrated T6SS gene cluster existed in the genome of porcine ExPEC strain PCN033 encoding three *hcp* genes

**Figure 1:**
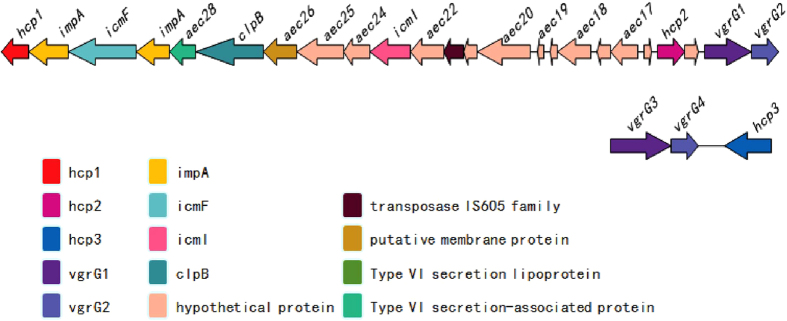
Schematic of the genetic organization of the porcine ExPEC PCN033 T6SS gene cluster. (**A**) ORFs in the T6SS are shown in different colours and directions. Sequence data for the cluster can be obtained from the National Centre for Biotechnology Information.

In total, the cluster contained 24 coding sequences, and three genes related to the T6SS occured outside the cluster (Fig. 1). Two *hcp* genes, *hcp1* and *hcp2*, were dispersed in the T6SS cluster, whereas *hcp3* was located at quite a distance from the cluster. Although the amino acid sequences of Hcp1, Hcp2 and Hcp3 shared some conversed sites, they also showed little mutual homology (Supplementary Figure S1), implying that they contributed in distinct ways to the pathogenesis of porcine ExPEC strain PCN033. We also found out they shared some conserved sites with Hcp proteins in other pathogens, including NMEC RS218, APEC TW-XM, EAEC 042, *P. aeruginosa* PA01, *V. cholerae* and *A. hydrophila* SSU (Supplementary Figure S1).

### Construction and growth characteristics of mutant strains Δ*hcp1*, Δ*hcp2*, Δ*hcp3* and Δ*hcp1*Δ*hcp2*Δ*hcp3*

**Figure 2:**
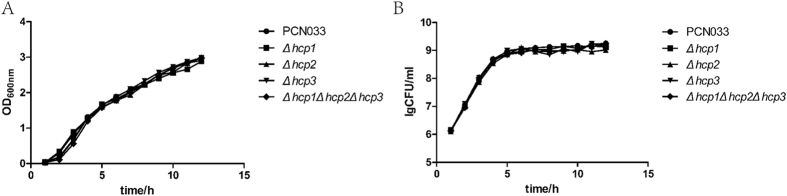
Growth characteristic of the parental and mutant strains. (**A**) Measurement results of OD_600 nm_ for mutant and parental strains. (**B**) CFU per ml of mutant and parental strains during 12 h generating time.

The Δ*hcp1*, Δ*hcp2*, Δ*hcp3* and Δ*hcp1*Δ*hcp2*Δ*hcp3* mutants constructed from porcine ExPEC strain PCN033 were confirmed with PCR using the primers listed in Table 1 (Supplementary Figure S2). The growth characteristics of the parental and mutant strains were determined. The mutant strains shared similar growth kinetics with the parental strain, indicating that the mutants did not have a growth defect (Fig. 2). Recombination plasmids used for complement strains were identified by dual-enzyme digestion in Supplementary Figure S3.

### Hcp family proteins contributed to bacterial competition

**Figure 3:**
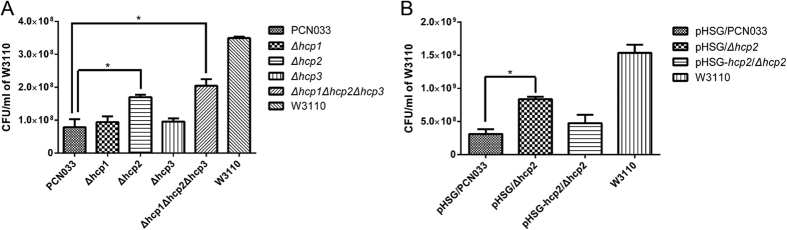
The role of Hcp family proteins in bacterial competition. (**A**) Final CFU/ml of *E. coli* K12 strain W3110 after competing with PCN033 and its derivatives. After 12 h generating at 30 °C, CFU/ml of W3110 was 3.54E + 08 ± 8.23E + 06. When competing with ExPEC strain PCN033, the CFU/ml of W3110 declined to 7.83E + 07 ± 4.31E + 07. CFU/ml of W3110 competing with Δ*hcp2* or Δ*hcp1*Δ*hcp2*Δ*hcp3* were 1.70E + 08 ± 1.25E + 07, 2.04E + 08 ± 3.46E + 07, respectively. The error bars indicated the SD of the means of three independent experiments. Statistically significant differences were indicated. *P ≤ 0.05. (**B**) The complement of *hcp2* can restore the bacterial competing with *E coli.* K12 strain W3110. The invasive CFU/ml of W3110 when competing with pHSG-*hcp3*/Δ*hcp3* was 4.75E + 08 ± 2.50E + 08, as pHSG/PCN033 and pHSG/Δ*hcp3* were 3.13E + 08 ± 1.44E + 08, 8.38E + 08 ± 7.50E + 07, respectively. The error bars indicated the SD of the means of three independent experiments. Statistically significant differences were indicated. *P ≤ 0.05.

In amount comparison of W3110 in different groups, lower amount of W3110 indicated stronger competitive ability of subject bacterial, while higher amount indicated weaker competitive ability. We initially compared two groups, Δ*hcp1*Δ*hcp2*Δ*hcp3* and the parental strain PCN033, and the Δ*hcp1*Δ*hcp2*Δ*hcp3* mutant showed a significantly reduced capacity for bacterial competition. We then compared Δ*hcp1*, Δ*hcp2* and Δ*hcp3* in the same way, as shown in Fig. 3A. Δ*hcp2* showed a marked decline in its competitive capacity and the defect was restored by recombination plasmid pHSG::*hcp2*, as shown in Fig. 3B; therefore, we inferred that Hcp2 in porcine ExPEC strain PCN033 was predominantly involved in its competition with *E. coli* K12 strain W3110.

### Hcp family proteins contributed to organ colonization by porcine ExPEC strain PCN033

**Figure 4:**
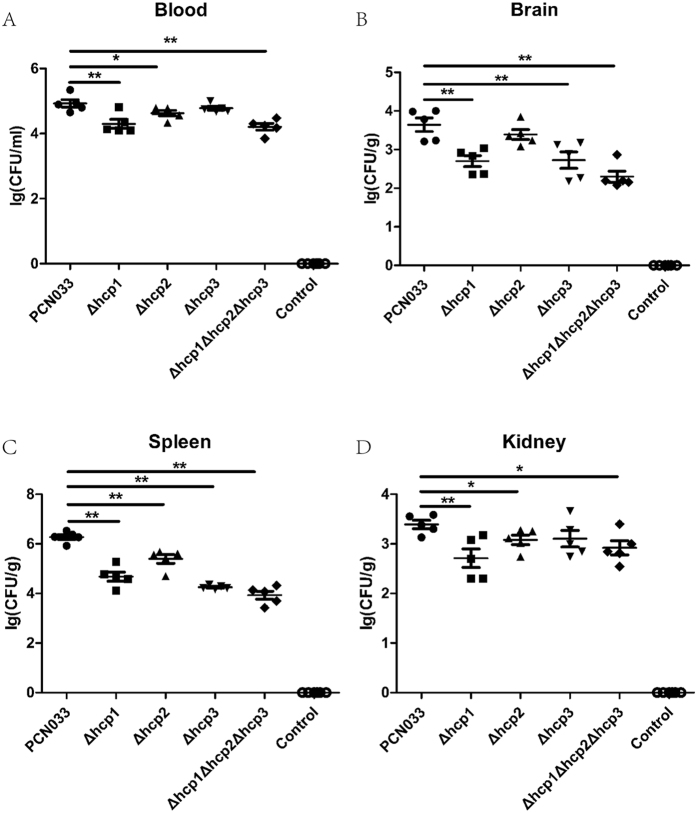
Colonization ability in different tissues of mice after challenged by porcine ExPEC strain PCN033 and mutant strains. Groups of five C57BL/6 mice were challenge via the caudal vein with 10^7^ CFU of parental and mutant strains. Heart perfusion was operated after 6 h. We examined bacterial concentrations in the blood (**A**), brain (**B**), spleen (**C**) and kidney (**D**) were examed. The error bars indicated the SD of the means of three independent experiments. Statistically significant differences were indicated. *P ≤ 0.05.

We estimate the colonization capacity of all the mutant strains in mouse organs (brain, kidney and spleen) and the degree of bacteraemia (Fig. 4). The bacteria in the blood decreased after the *hcp* genes were deleted and the colonization capacity of the mutants in the brain, spleen and kidney was also significantly attenuated.

### Hcp family proteins modulated the bacterial interaction with PK-15 cells during adherence and invasion

**Figure 5:**
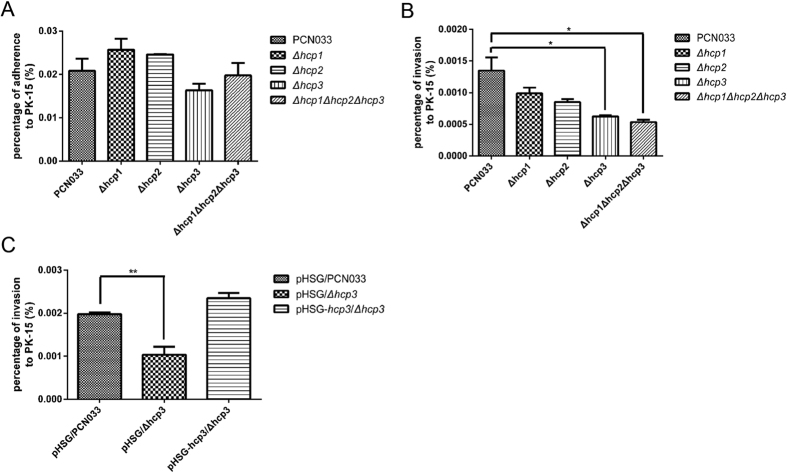
Adherence and invasion assays performed in PK-15 cell model. Adherence and invasion capacities were expressed in adherence and invasion percentage for each strain. (**A,B**) showed adherence and invasion assays, respectively, in PK-15 cell model; C showed *hcp3* can restore invasion ability in PK-15cell model. (**A**) showed that adherence capacities of all mutants were not significantly lower than that of the parental strain. (**B**) showed that the Δ*hcp3* mutant and the Δ*hcp1*Δ*hcp2*Δ*hcp3* mutant had lower invasion capacities for PK-15 cell than that of the parental strain, with invasion percentage of 0.000624 ± 0.0000284 and 0.000537 ± 0.0000621, respectively. (**C**) Showed invasion percentage of pHSG/PCN033 was 0.00198 ± 0.000074, pHSG/Δ*hcp3* 0.00104 ± 0.00032, pHSG-*hcp3*/Δ*hcp3* 0.00268 ± 0.000785. *hcp3* restored invasion capability of strain. The error bars indicated the SD of the means of three independent experiments. Statistically significant differences were indicated. *P ≤ 0.05.

To determine whether Hcp1, Hcp2 and Hcp3 play roles in porcine ExPEC adherence and invasion of mammalian cells, we chose PK-15 cells as the cell model. The Hcp proteins were not involved in bacterial adherence to PK-15 cells (Fig. 5A), while the invasive capacity of the bacterium was influenced by the Hcps to different degrees (Fig. 5B). Mutants of Δ*hcp3* and Δ*hcp1*Δ*hcp2*Δ*hcp3* showed significant decline of the invasive capacity comparing with that of parental strain in PK-15 cells. In comparison, group of Δ*hcp3* displayed highest reduction among the three group. To further confirm the predominance of *hcp3* in the invasion, we constructed complement strain of pHSG-*hcp3*/Δ*hcp3* to perform invasion experiment in PK-15 model. The result indicated that rescue of *hcp3* could recover their invasion capability (Fig. 5C). This suggested that the Hcp proteins mainly influence the invasive capacity of porcine ExPEC strain PCN033, though did not contribute to its adherence. And in invasion, Hcp3 played a predominant role.

### Hcp family proteins enhanced the PCN033 intracellular replication ability in porcine alveolar macrophages (PAMs)

**Figure 6:**
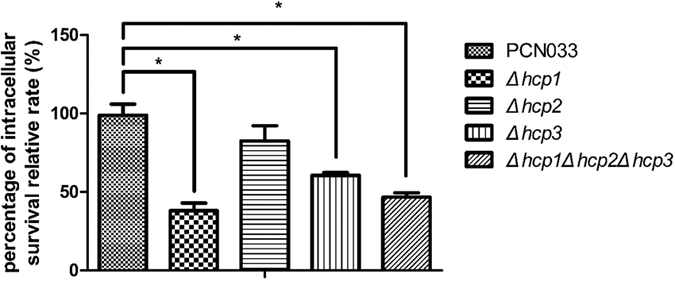
Bacterial reproduction in PAMs by the *hcp*s deletion. The intracellular reproductive rates were expressed as frequencies relative to that of the parental strain. The rates of Δ*hcp1*, Δ*hcp3* and the triple mutant Δ*hcp1*Δ*hcp2*Δ*hcp3* were 38.1 ± 8.3%, 60.6 ± 2.9% and 46.6 ± 4.9% (mean ± SD), respectively, relative to the frequency of parental strain, which was arbitrarily set at 100%. The error bars indicated the SD of the means of three independent experiments. Statistically significant differences were indicated. *P ≤ 0.05.

We chose PAMs as the cell model, as the parental strain was isolated from swine. An intracellular replication assay was performed as described below^20^. Δ*hcp1*, Δ*hcp3* and Δ*hcp1*Δ*hcp2*Δ*hcp3* showed significantly reduced abilities to replicate intracellularly, retaining 38.1%, 60.6% and 46.6% of replication capacity of parental strain, respectively, whereas the Δ*hcp2* mutation had no effect, with replication equivalent to that of the parental strain (Fig. 6).

## Discussion

Many bacterial genomes had several T6SS clusters or multiple copies of *hcp* genes. Hcp proteins contribute to the pathogenesis of many bacteria, including *V. cholerae, A. hydrophila, P. aeruginosa* and *B. pseudomallei*^4^,^5^,^6^,^7^. Porcine ExPEC strain PCN033 was isolated from the brain of a swine with meningitis in China^21^,^22^. As the pathogenic mechanism remained unclear, we sequenced the whole genome of the parental strain and found a putative integrated T6SS gene cluster in its chromosome^22^. In that T6SS cluster, three *hcp* family genes were identified. However, the roles of the Hcp proteins of porcine ExPEC PCN033 were largely unknown. The open reading frames (ORFs) of the *hcp* genes showed negligible similarity to those of other bacteria, but the corresponding amino acid sequences were conserved at some sites relative to those of other species(Supplementary Figure S1). According to a multiple sequence alignment, the amino acid sequences of Hcp1, Hcp2 and Hcp3 showed mutual similarity at some sites. A BLAST search of the National Center for Biotechnology Information database showed that Hcp1, Hcp2 and Hcp3 of ExEPC strain PCN033 shared relatively high homology with Hcp2 of NMEC strain RS218, the Hcps of APEC strain TW-XM, Hcp of *A. hydrophila* and Hcp3 of *P. aeruginosa*^4^,^5^,^11^,^12^. Hcp1 of porcine ExPEC strain PCN033 also shared some amino acid sites with Hcp1 of *V. cholerae*, and Hcp2 had almost the same sequence as Hcp2 of NMEC strain RS218 and XmtR of APEC strain TW-XM, with only nine amino acid differences^11^,^12^,^15^. The former half of Hcp3 shared some conserved amino acids with other Hcp proteins, whereas the other half showed no homology. We speculated that the Hcps of ExPEC strains share some analogous function, but as the conservation between them was not that close, they might have diverse functions. In this study, we first constructed the Δ*hcp1*Δ*hcp2*Δ*hcp3* mutant strain and performed a bacterial competition assay, which demonstrated a significant reduction in competition compared with that of the parental strain. Because bacterial competition was an important function of the T6SS, we confirmed that the T6SS in porcine ExPEC strain PCN033 was functional. To determine the individual role of each Hcp, mutant strains Δ*hcp1*, Δ*hcp2* and Δ*hcp3* were constructed and assessed for their capacities for bacterial competition, colonization, cell adherence and invasion, as well as intracellular reproduction.

After we confirmed that the duelling ability of Δ*hcp1*Δ*hcp2*Δ*hcp3* was lower than that of the parental strain, we assessed the abilities of the individual mutant strains Δ*hcp1*, Δ*hcp2* and Δ*hcp3* to compete with *E. coli* K12 strain W3110. Hcp2 seemed to play the major role in inter-bacterial competition, suggesting that it might be an effector of T6SS. Utilizing SecReT6 (http://db-mml.sjtu.edu.cn/SecReT6/) to analyze the T6SS of ExPEC PCN033, we found besides Hcp family protein, other T6SS effectors were presented including three Rhs family (Supplementary Table S1) which might be mainly involved in bacterial competition. Having a growth advantage over other bacteria increased the survival of the pathogen would survive in a natural habitat. Thus, we believed that this ability allowed porcine ExPEC strain PCN033 to better compete with other pathogens. After we confirmed that the Hcp family proteins contributed to bacterial competition, we anticipated that they also had other functions in pathogenesis. Therefore, we investigated the capacity for colonization of each strain in mice. Bacteraemia decreased significantly after the *hcp* genes were deleted, and the bacterial concentrations in important organs declined markedly. The Hcp family proteins might influence the quantities of bacteria in the organs in two ways: reducing bacteraemia and weakening the colonization ability of the bacterium. Hcp was not involved in the virulence of *V. cholerae* in an infant mouse model, but the Hcp protein of APEC SEPT362 was involved in its virulence^9^,^15^. The Hcp proteins of porcine ExPEC strain PCN033 shared some conserved sites with those two Hcps, but the Hcp proteins were likely to function in diverse ways in different pathogens. The interaction of porcine ExPEC PCN033 with its host was a multifactorial and complex phenomenon, and many other virulence factors are involved, such as IutA and KpsMII^22^,^23^.

The Hcp family proteins were also involved in the bacterial interaction with distinct cell models^10^,^11^,^12^. Here, we demonstrated that they functioned both in adherence and invasion when interacting with an epithelial cell model (PK-15 cells), and in their survival rate after their phagocytosis in PAMs. We found none of the three Hcp family proteins contributed to the adherence process, but all of them played a part in the invasion process, with Hcp3 functioning predominantly. In APEC TW-XM, XmtR had nearly the same sequence as Hcp2 of PCN033, but it was not known whether it was involved in bacterial invasion. Our findings suggested that Hcp1, Hcp2 and Hcp3 of porcine ExPEC strain PCN033 were all associated with its interaction with mammalian cells, but in different stages of the interaction process. However, the mechanism involved in the adherence and invasion of this strain required further investigation. In the intracellular replication assay, our results suggested that Hcp1 and Hcp3 contribute to its intracellular survival, whereas Hcp2 did not seem to be involved in this process. In *A. hydrophila*, Hcp modulated the immune response^4^. Although Hcp2 in PCN033 had some homology to Hcp2 of *A. hydrophila*, their functions were mostly likely to differ in terms of the immune response, which might due to pathogen specificity. In the interaction with eukaryotic cells, Hcps may play roles directly, or they could push other effectors to take effect.

In this study, we had shown for the first time that the Hcp family proteins play functional roles in porcine ExPEC strain PCN033, demonstrating that the T6SS was fully functional in this pathogen. Three different *hcps* were detected in porcine ExPEC strain PCN033, and Hcp1, Hcp2 and Hcp3 had diverse functions in its interactions with bacteria and eukaryotic cells, and the Hcp family proteins all seemed to function in the pathogenesis of porcine ExPEC strain PCN033 in the mouse model. Interestingly, *hcp3*, which located distantly from the T6SS cluster on the porcine ExPEC strain PCN033 chromosome, clearly influenced PCN033. Hcp2 predominantly contributed to bacterial competition, whereas Hcp1 and Hcp3 were mainly involved in its interaction with host cells. In a mouse model, all the Hcp family proteins contributed to bacterial colonization *in vivo*. Although *hcp1, hcp2, hcp3* shared some conserved sites, they also had diverse amino acid sequence, they might have diverse function.

## Materials and Methods

### Bacterial strains and culture conditions

Porcine ExPEC strain PCN033 (O11:K2), belonging to *E. coli* reference (ECOR) group D, was isolated from a pig with meningitis in China^21^,^22^. The strain was shown to cause pathological symptoms in a pig model^22^. The bacteria were grown routinely in LB or on solid medium containing 1.5% agar at 37 °C with or without shaking. When necessary, the LB medium was supplemented with kanamycin at 50 mg/mL. The complement strains were incubated in LB medium with chloramphenicol at 50 mg/mL. To accomplish multiple sequence alignment of amino acid sequences of Hcps in different pathogens, Clustal W (http://www.ebi.ac.uk/Tools/msa/clustalo/) was used and Boxshade (http://www.ch.embnet.org/software/BOX_form.html) to highlight the conservative sites.

To infer effectors of T6SS in ExPEC strain PCN033, we blasted in a website, namely http://db-mml.sjtu.edu.cn/SecReT6/.

### Construction of mutant strains Δ*hcp1*, Δ*hcp2*, Δ*hcp3* and Δ*hcp1*Δ*hcp2*Δ*hcp3* and their complement strains

The markerless mutant strains Δ*hcp1*, Δ*hcp2*, Δ*hcp3* and Δ*hcp1*Δ*hcp2*Δ*hcp3* were constructed using the double-selection strategy of allelic exchange mutagenesis with the suicide vector pRE112, as described below^24^. We deleted 483 bp of the *hcp1*, 519 bp of *hcp2* and 1149 bp of *hcp3*, thus obtaining mutants from which the complete ORF was removed. The upstream and downstream regions of *hcp1, hcp2* and *hcp3* were separately amplified with PCR from the genomic DNA of porcine ExPEC strain PCN033 with primers P1/P2, P3/P4, P5/P6, P7/P8, P9/P10 and P11/P12. The purified PCR products were sequenced and ligated into pRE112 to construct pRE::*hcp1*, pRE::*hcp2* and pRE::*hcp3*, lacking the ORFs of the target genes. The reconstructed plasmids were introduced into *E. coli* strain x7213 and delivered into strain PCN033 through conjugation transfer when the bacteria were co-cultured. The transconjugants containing the reconstructed plasmids were integrated into the PCN033 chromosome by single-crossover events and were selected on LB agar containing chloramphenicol. Allelic exchange between the chromosomal gene and the mutagenized plasmid copy was achieved by a second crossover event counter-selected on LB agar containing 10% sucrose to determine the excision of the reconstructed suicide vector from the chromosome. Mutant Δ*hcp1*Δ*hcp2*Δ*hcp3* was constructed after Δ*hcp1*Δ*hcp3* was successfully constructed. The Δ*hcp1*, Δ*hcp2*, Δ*hcp3* and Δ*hcp1*Δ*hcp2*Δ*hcp3* mutants were confirmed by their ability to grow on LB agar plates and their inability to grow with chloramphenicol, and the final deletions were confirmed with PCR using primers T1/T2, T3/T4, T5/T6, T7/T8, T9/T10 and T11/T12, as described previously^25^. Primer T1/T2, T5/T6 and T9/T10 included the target gene’s upstream and downstream homologue arms, whereas T3/T4, T7/T8 and T11/T12 bound inside the target gene’s ORF. The correct deletion was verified by sequencing the resulting PCR product.

Complement strains were constructed by using recombination plasmids pHSG::*hcp2* and pHSG::*hcp3*. The complete coding region of *hcp2, hcp3* were amplified by using primers T7/T8 and T11/T12 and inserted into pHSG396 to generate recombination plasmids pHSG::*hcp2*, pHSG::*hcp3*., which were then introduced into Δ*hcp2* and Δ*hcp3* strain respectively using electrotransformation instruments (Bio-Rad, USA). We also introduce plasmid pHSG396 into mutant strains separately to perform experiment in order to eliminate polar effect of the plasmid. The complement strains were selected on LB agar medium containing chloramphenicol at 50 mg/mL.

The complete genome sequence of porcine ExPEC PCN033 has been submitted to the National Center for Biotechnology Information GenBank under accession number NZ-CP006632.1. All the bacteria and plasmids used in this study were listed in Table 2, while all primers in Table 1.

### Determination of growth characteristics

After overnight culture, porcine ExPEC strain PCN033 and the mutant strains were diluted 1:1000 and grown in LB medium at 37 °C with steady shaking. Their growth characteristics within 12 h were determined with optical density measurements at 600 nm (OD_600_), and CFU/h was determined at the appropriate dilutions on LB agar plates after overnight culture at 37 °C.

### Bacterial competition assay

The growth competition assay was performed using porcine ExPEC PCN033 and its derivatives as the predators, and the *E. coli* K12 W3110 strain as the prey, as described previously^26^, with minor adjustments. The bacteria were grown in LB medium to an OD_600_ of 1 at 37 °C with shaking, adjusted to OD_600_ 0.5 with phosphate-buffered saline (PBS), and mixed to a prey:predator ratio (CFU) of 10:1. Then, 25 μL of the mixture was spotted onto nitrocellulose membrane (Millipore, USA) on pre-warmed LB agar at 30 °C for 12 h, and the CFU in 25 μL of prey was used as the background value. The bacterial spots were harvested, and the CFU/mL values of the surviving predator and prey were determined by plating 10-fold serial dilutions on selective medium plates, because the predators were resistant to kanamycin but the prey were not. All the competition data are expressed with the amount of survival prey. All assays were repeated three times in triplicate.

### Cell culture

Porcine alveolar macrophages were obtained from the lungs of 3- to 4-week-old *E. coli.*-free piglets with sterile bronchoalveolar lavage, as described previously^20^. The isolated PAMs were cultured in Dulbecco’s modified Eagle’s medium (DMEM; Gibco) containing 10% (v/v) heat-inactivated foetal bovine serum (FBS; Gibco), 100 U/mL penicillin and 100 μg/mL streptomycin (Sigma). Porcine kidney epithelial cells (PK-15) were cultured in DMEM containing 10% (v/v) heat-inactivated FBS. All cells were cultured at 37 °C in a humidified incubator with 5% CO_2_.

### Adherence and invasion assays

The adherence and invasion assays were performed as previously described^27^, with minor adjustments, using PK-15 cells as the cell model. Briefly, 24-well tissue culture plates were seeded with confluent monolayer. Each bacterial strain was added at a multiplicity of infection (MOI) of 100 and the plates were incubated for 90 min to allow the *Escherichia*-model cell interaction. For the adherence assay, after incubation, the cells were vigorously washed five times with PBS (Hyclone) to eliminate nonspecific bacterial adherence. They were then lysed by incubation in sterile water at room temperature for 15 min, and after homogenization, 10-fold serial dilutions were plated onto LB agar plates to determine the total CFU. For the invasion assay, after incubation, the cell monolayers were washed three times with PBS, and then incubated with medium containing 25 μg/mL cefotaxime for 1 h to kill any extracellular bacteria. The number of bacteria that had invaded the cells was counted as described above. All the assays were performed three times in triplicate.

### Intracellular replication assay

The intracellular replication assay was performed as described previously^27^, with some adjustments. PAMs were seeded in 24-well tissue culture plates and 2 h before infection, the non-adherent cells were washed off with PBS. *Escherichia coli* were added to the PAMs at an MOI of 10. One hour after infection, medium containing 25 μg/mL cefotaxime was used to kill the extracellular bacteria. After another 1 h, the medium was replaced with fresh medium containing 10 μg/mL cefotaxime. The number of viable intracellular bacteria was assessed 3 h after infection, as described above. All the assays were performed in triplicate.

### Mouse model of bacterial colonization

Animal experiments were approved by the Laboratory Animal Monitoring Committee of Huazhong Agricultural University (No. HZAUMO-2014–010) and were performed in accordance with the recommendations in the Guide for the Care and Use of Laboratory Animals of Hubei Province, China. Thirty 8-week-old female C57BL/6 mice (Hubei Center for Disease Control and Prevention, Hubei, China) were randomly assigned to six groups. The mice were inoculated intravenously in the lateral tail vein with an infectious dose of 10^7^ CFU of bacteria in 0.9% NaCl. Six hours later, the mice were airway medicated to establish anaesthesia and their chests were opened. Blood from the right ventricle was collected to calculate the CFU of bacteria surviving in the blood. The mice were then perfused with a 1-mm needle inserted into the left ventricle of the heart with smooth force. The composition of the perfusate was 0.9% NaCl containing a final concentration of 10 U/L heparin sodium. After perfusion for 30 min, the mice were killed with cervical dislocation and their organs (brains, kidneys and spleens) were removed, weighed, homogenized and cultured to determine the CFU. Bacterial survival in the blood was expressed as CFU/mL, and bacterial colonization of the organs was expressed as CFU/g tissue, as described previously^28^.

### Statistical analysis

All data were evaluated with the Student’s *t* test using the SPSS ver. 19.0 software (SPSS Inc., Chicago, IL, USA). A P value of less than 0.05 was considered significant.

## Additional Information

**How to cite this article**: Peng, Y. *et al*. Roles of Hcp family proteins in the pathogenesis of the porcine extraintestinal pathogenic *Escherichia coli* type VI secretion system. *Sci. Rep.* **6**, 26816; doi: 10.1038/srep26816 (2016).

## Supplementary Material

Supplementary Information

## Figures and Tables

**Table 1:** Strains and plasmids used in this study.

**Strains**	**Description and Genotype**	**Source**
PCN033	ExPEC O11, phylogenetic group D, field strain from pig in China, Km^R^, Cm^S^	This study
Δ*hcp1*	PCN033 derivate, all frame deletion of *hcp1*, Km^R^,Cm^S^	This study
Δ*hcp2*	PCN033 derivate, all frame deletion of *hcp2*, Km^R^,Cm^S^	This study
Δ*hcp3*	PCN033 derivate, all frame deletion of *hcp3*, Km^R^,Cm^S^	This study
Δ*hcp1*Δ*hcp2*Δ*hcp3*	PCN033 derivate, all frame deletion of *hcp1, hcp2, hcp3*, Km^R^,Cm^S^	This study
pHSG-*hcp2*/Δ*hcp2*	Δ*hcp2* strain containing pHSG::*hcp2*, Km^R^, Cm^R^	This study
pHSG-*hcp3*/Δ*hcp3*	Δ*hcp3* strain containing pHSG::*hcp3*, Km^R^, Cm^R^	This study
pHSG/PCN033	PCN033 strain containing pHSG, Km^R^, Cm^R^	This study
pHSG/Δ*hcp2*	Δ*hcp2* strain containing pHSG, Km^R^, Cm^R^	This study
pHSG/Δ*hcp3*	Δ*hcp3* strain containing pHSG, Km^R^, Cm^R^	This study
x7213	Conjugation transfer host for recombinant vector	26
W3110	F-, lambda- IN(rrnD-rrnE)1 rph-1, Km^S^	23
**Plasmids**		
pRE112	pGP704 suicide plasmid, pir dependent, oriT, oriV, sacB, Cm^R^	26
pRE112::*hcp1*	Recombinant vector with the pRE112 background, designed for knockout of *hcp1*	This study
pRE112::*hcp2*	Recombinant vector with the pRE112 background, designed for knockout of *hcp2*	This study
pRE112::*hcp3*	Recombinant vector with the pRE112 background, designed for knockout of *hcp3*	This study
pHSG396	ori lacZ Cm^R^	Takara Bio
pHSG::*hcp2*	Complement vector of *hcp2*	This study
pHSG::*hcp3*	Complement vector of *hcp3*	This study

**Table 2:** Primers used in this study.

**Primer**	**Sequence (5′-3′)**	**Product size (bp)**	**Target product**
P1	ATGGTACCTGTTATGTTTTGTTCTCGCTT *KpnI*	817	upstream of *hcp1*
P2	CGGAATTCTTTACTGTAATAACATAATAAATTACTATT *EcoRI*		
P3	CGGAATTCGATAATCTCCTTTAATTAAATAATTATTTT *EcoRI*	815	downstream of *hcp1*
P4	ATGAGCTCCGGCAATATTCTGCGTCA *SacI*		
P5	ATGGTACCATATTCAGCGCCACATTCAGT *KpnI*	1000	upstream of *hcp2*
P6	CGGAATTCTTGTAAACTCCTTGTTAACGTGG *EcoRI*		
P7	CGGAATTCGTTAAGCCAACAGCATCCG *EcoRI*	1000	downstream of *hcp2*
P8	ATGAGCTCTTGAAACGTCCGGGGTAG *SacI*		
P9	ATGGTACCGTCATCACCATCGAAACCTC *KpnI*	801	upstream of *hcp3*
P10	GGCTCGAGAACTGAAATTAACGTACTG *XhoI*		
P11	CACTCGAGCCTTGAAAAACCTTTTG *XhoI*	810	downstream of *hcp3*
P12	ATGAGCTCCGCAACGGCGGCGATGTTC *SacI*		
T1	TTATTGAGCTGAGTATTTTTTTTGC	985/513	a fragment containing *hcp1*
T2	TAACCCTTTCCTATATCAAAAATGTC		
T3	ATGGCAAATATGAGTTATTTATCTTT	483	an internal fragment of *hcp1*
T4	TTACTGTACACGATCCTGCCA		
T5	TTATGGAGGCAATACGGACTT	1019/500	a fragment containing *hcp2*
T6	AGGCCGTCCACTTCCAG		
T7	CCGCTCGAGATGCCAACACCGTGTTATATCT *XhoI*	519	an internal fragment of *hcp2* and complement for *hcp2*
T8	CGGGGTACCTTATGCTTCCAGCGGTGC *KpnI*		
T9	GTATCCCTTTTCAGTAAC	1593/444	a fragment containing *hcp3*
T10	CAATGGCAGACACCTTAG		
T11	CCGCTCGAGATGAGTGATATTATTTACCTGAACATAA *XhoI*	1149	an internal fragment of *hcp3* and complement for *hcp3*
T12	CGGGGTACCTCAGTTATCATAGTCAAGTGCATC *KpnI*		

## References

[b1] Pukatzki S. . Identification of a conserved bacterial protein secretion system in *Vibrio cholera* using the Dictyostelium host model system. Proc Natl Acad Sci USA 103(5), 1528–1533 (2006). 16432199 10.1073/pnas.0510322103 PMC1345711

[b2] Basler M., Pilhofer M., Henderson G. P., Jensen G. J. & Mekalanos J. J. Type VI secretion requires a dynamic contractile phage tail-like structure. Nature. 483, 182–187 (2012). 22367545 10.1038/nature10846 PMC3527127

[b3] Pukatzki S., McAuley S. B. & Miyata S. T. The type VI secretion system: translocation of effectors and effector-domains. Curr Opin Micro. 12(1), 11–7 (2009). 10.1016/j.mib.2008.11.010 19162533

[b4] Suarez G., Sierra J. C., Kirtley M. L. & Chopra A. K. Role of Hcp, a type 6 secretion system effector, of *Aeromonas hydrophilain* modulating activation of host immune cells. Microbiology. 156, 3678–88 (2010). 20798163 10.1099/mic.0.041277-0 PMC3068704

[b5] Mougous J. D. . A Virulence Locus of *Pseudomonas aeruginosa* Encodes a Protein Secretion Apparatus. Science. 312, 1526–30 (2006). 16763151 10.1126/science.1128393 PMC2800167

[b6] Burtnick M. N. . The Cluster 1 Type VI Secretion System Is a Major Virulence Determinant in *Burkholderia pseudomallei*. Infect Immun. 79(4), 1512–25 (2011). 21300775 10.1128/IAI.01218-10 PMC3067527

[b7] Fu Y., Waldor M. K. & Mekalanos J. J. Tn-Seq Analysis of *Vibrio cholera* Intestinal Colonization Reveals a Role for T6SS-Mediated Antibacterial Activity in the Host. Cell Host Microbe. 14(6), 652–63 (2013). 24331463 10.1016/j.chom.2013.11.001 PMC3951154

[b8] Ma L. S., Hachani A., Lin J. S., Filloux A. & Lai E. M. *Agrobacterium tumefaciens* deploys a superfamily of type VI secretion DNase effectors as weapons for interbacterial competition in planta. Cell Host Microbe. 16(1), 94–104 (2014). 24981331 10.1016/j.chom.2014.06.002 PMC4096383

[b9] de Pace F. . The Type VI Secretion System Plays a Role in Type 1 Fimbria Expression and Pathogenesis of an Avian Pathogenic *Escherichia coli* Strain. Infect Immun. 78(12), 4990 (2010). 20855516 10.1128/IAI.00531-10 PMC2981326

[b10] de Pace F. . Characterization of IcmF of the type VI secretion system in an avian pathogenic *Escherichia coli* (APEC) strain. Microbiology. 157, 2954–62 (2011). 21778203 10.1099/mic.0.050005-0 PMC3353391

[b11] Ma J. . Two Functional Type VI Secretion Systems in Avian Pathogenic *Escherichia coli* Are Involved in Different Pathogenic Pathways. Infect Immun. 82(9), 3867–79 (2014). 24980972 10.1128/IAI.01769-14 PMC4187841

[b12] Zhou Y. . Hcp Family Proteins Secreted via the Type VI Secretion System Coordinately Regulate *Escherichia coli* K1 Interaction with Human Brain Microvascular Endothelial Cells. Infect Immun. 80(3), 1243–51 (2012). 22184413 10.1128/IAI.05994-11 PMC3294675

[b13] Brunet Y. R., Espinosa L., Harchouni S., Mignot T. & Cascales E. Imaging Type VI Secretion-Mediated Bacterial Killing. Cell Rep. 3(1), 36–41 (2013). 23291094 10.1016/j.celrep.2012.11.027

[b14] Wu H. Y., Chung P. C., Shih H. W., Wen S. R. & Lai E. M. Secretome Analysis Uncovers an Hcp-Family Protein Secreted via a Type VI Secretion System in *Agrobacterium tumefaciens*. J Bacteriol. 190(8), 2841–50 (2008). 18263727 10.1128/JB.01775-07 PMC2293243

[b15] Williams S. G., Varcoe L. T., Arrridge S. R. & Manning P. A. *Vibrio cholera* Hcp, a secreted protein coregulated with HlyA. Infect Immun. 64(1), 283–9 (1996). 8557353 10.1128/iai.64.1.283-289.1996 PMC173757

[b16] Ballister E. R., Lai A. H., Zuckermann R. N., Cheng Y. & Mougous J. D. In vitro self-assembly of tailorable nanotubes from a simple protein building block. Proc Natl Acad Sci USA 105(10), 3733–3738 (2008). 18310321 10.1073/pnas.0712247105 PMC2268831

[b17] Silverman J. M. . Haemolysin Coregulated Protein Is an Exported Receptor and Chaperone of Type VI Secretion Substrates. Mol Cell. 51, 584–93 (2013). 23954347 10.1016/j.molcel.2013.07.025 PMC3844553

[b18] Koskiniemi S. . Rhs proteins from diverse bacteria mediate intercellular competition. Proc Natl Acad Sci USA. 110(17), 7032–7 (2013). 23572593 10.1073/pnas.1300627110 PMC3637788

[b19] Decoin V. . A Type VI Secretion System Is Involved in Pseudomonas fluorescens Bacterial Competition. Plos one. 9(2), e89411 (2014). 24551247 10.1371/journal.pone.0089411 PMC3925238

[b20] Boyen F. . Role of SPI-1 in the interactions of *Salmonella Typhimurium* with porcine macrophages. Vet Microbiol. 113(1–2), 35–44 (2006). 16310983 10.1016/j.vetmic.2005.10.018

[b21] Tan C. . Genome Sequence of a Porcine Extraintestinal Pathogenic *Escherichia coli* Strain. J Bacterial. 193(18), 5038 (2011). 10.1128/JB.05551-11 PMC3165708 21742868

[b22] Liu C. . Genome analysis and in vivo virulence of porcine extraintestinal pathogenic *Escherichia coli* strain PCN033. BMC Genomic. 16(1), 717 (2015). 10.1186/s12864-015-1890-9 PMC4578781 26391348

[b23] Teng C. H. . *Escherichia coli* K1 RS218 Interacts with Human Brain Microvascular Endothelial Cells via Type 1 Fimbria Bacteria in the Fimbriated State. Infect Immun. 73(5), 2923–31 (2005). 15845498 10.1128/IAI.73.5.2923-2931.2005 PMC1087349

[b24] Edwards R. A., Keller L. H. & SchiVerli D. M. Improved allelic exchange vectors and their use to analyze 987P fimbria gene expression. Gene. 207(2), 149–57 (1998). 9511756 10.1016/s0378-1119(97)00619-7

[b25] Hou B. . TolC promotes ExPEC biofilm formation and curli production in response to medium osmolarity. Biomed Res Int. http://dx.doi.org/10.1155/2014/574274 (2014). 10.1155/2014/574274 PMC4163439 25243151

[b26] Gueguen E. & Cascales E. Promoter Swapping Unveils the Role of the Citrobacter rodentium CTS1 Type VI Secretion System in Interbacterial Competition. Appl Environ Microbiol. 79(1), 32–38 (2012). 23064344 10.1128/AEM.02504-12 PMC3536073

[b27] Frandoloso R., Martı´nez-Martı´nez S., Gutie´rrez-Martı´n C. B. & Rodrı´guez-Ferri E. F. *Haemophilus parasuis* serovar 5 Nagasaki strain adheres and invades PK-15 cells. Vet Microbiol. 154 **(3–4)**, 347–52 (2012). 21839589 10.1016/j.vetmic.2011.07.022

[b28] Zhu L. . Arachidonic Acid Metabolism Regulates *Escherichia coli* Penetration of the Blood-Brain Barrier. Infect Immun. 78(10), 4302–10 (2010). 20696828 10.1128/IAI.00624-10 PMC2950368

