# Supplementary material for: Roles of Hcp family proteins in the pathogenesis of the porcine extraintestinal pathogenic Escherichia coli type VI secretion system
Source: Sci Rep. 2016 May 27;6:26816. doi: 10.1038/srep26816 (PMC4882540; doi:10.1038/srep26816)
Supplement: Supplementary Information [file srep26816-s1.doc]

**Supplementary Information for**

**Roles of Hcp family proteins in the pathogenesis of the porcine extraintestinal pathogenic *Escherichia coli* type VI secretion system**

Ying Peng^1,2^, Xiangru Wang^1,2^, Jin Shou^1,2^, Bingbing Zong^1,2^, Yanyan Zhang^1,2^, Jia Tan^1,2^，Jing Chen^1,2^, Linlin Hu^1,2^, Yongwei Zhu^2^, Huanchun Chen^1,2^, Chen Tan^1,2,*^

^1^State Key Laboratory of Agricultural Microbiology, College of Veterinary Medicine, Huazhong Agricultural University, Wuhan, Hubei, 430070, China;

^2^The Cooperative Innovation Center for Sustainable Pig Production, Key Laboratory of development of veterinary diagnostic products of Ministry of Agriculture, Huazhong Agricultural University, Wuhan, Hubei, 430070, China;

*** Corresponding author**. Phone: +86-27-87287170. Fax: +86-27-87282608.

E-mail address: tanchen@mail.hzau.edu.cn

**Supplementary Figure S1.** **Amino acid sequence alignment of Hcp1, Hcp2 and Hcp3 of porcine ExPEC PCN033 and Hcps in other pathogens.**


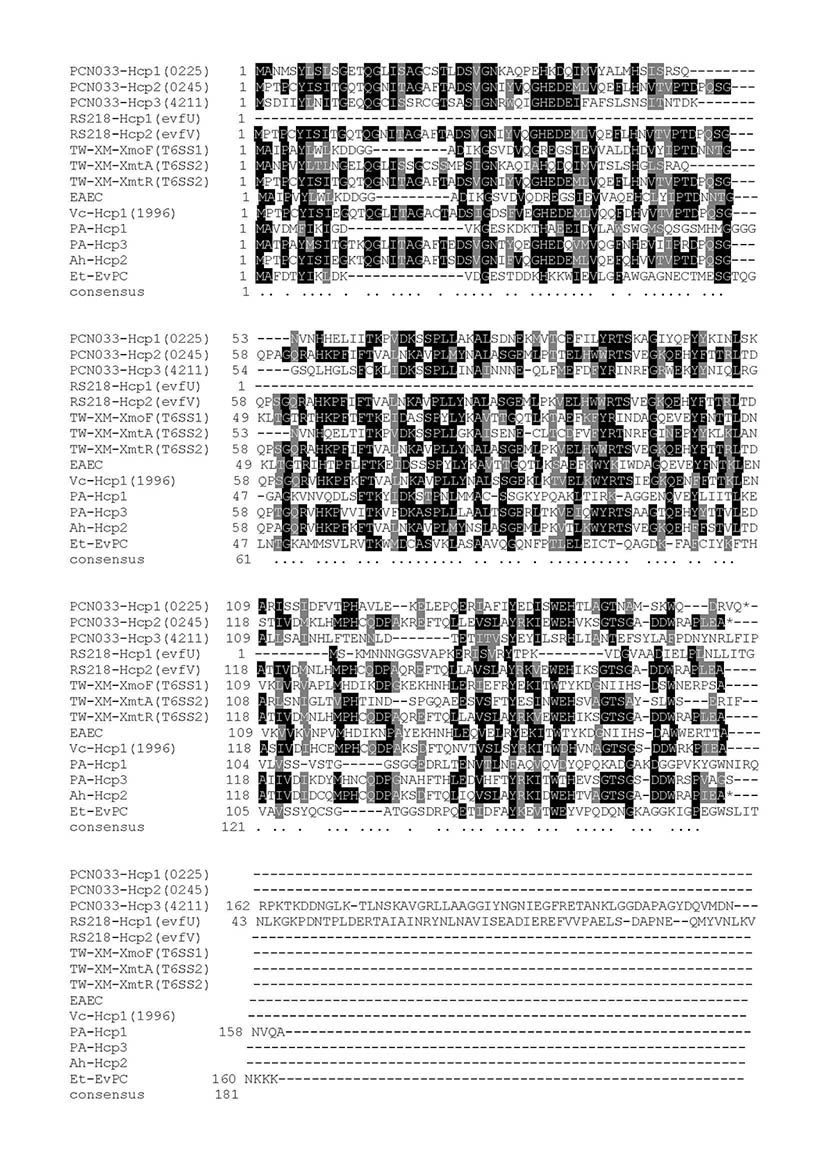


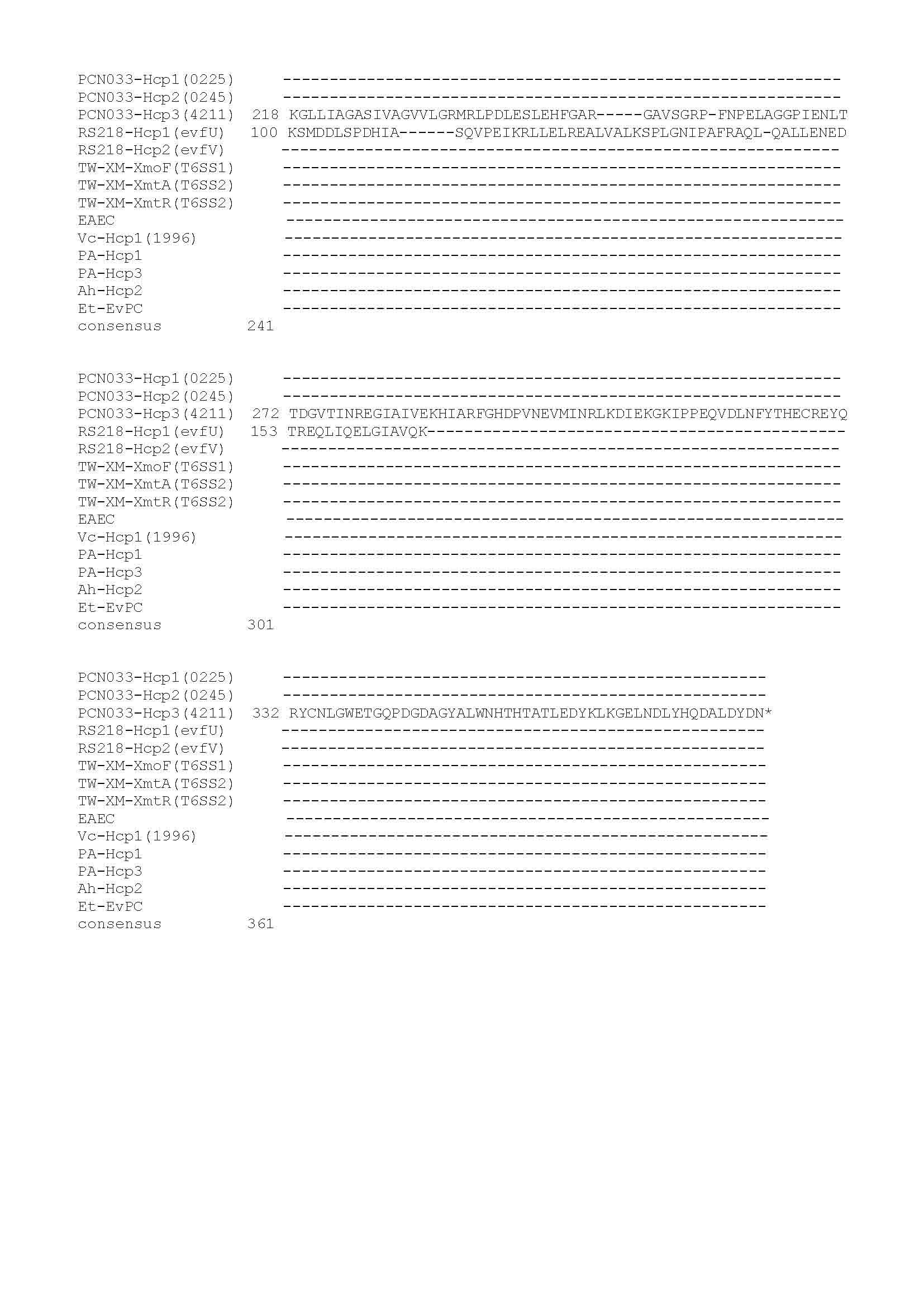


**Supplementary Figure S2. PCR identification of mutants Δ*hcp1*, Δ*hcp2*, Δ*hcp3* and Δ*hcp1*Δ*hcp2*Δ*hcp3* constructed from porcine ExPEC PCN033.** A: Principle for identifying the four mutants by using primers T1/T2, T3/T4, T5/T6, T7/T8, T9/T10 and T11/T12. B: PCR identification of mutant strains. The genomes used in the PCR were indicated above the lane. Primers T1/T2 (lane1, 3, 13, 19), T3/T4 (lane 2, 4, 14, 20), T5/T6 (lane 5, 7, 15, 21), T7/T8 (lane 6, 8, 16, 22), T9/T10 (lane 9, 11, 17, 23) and T11/T12 (lane 10, 12, 18, 24) were used for identification.


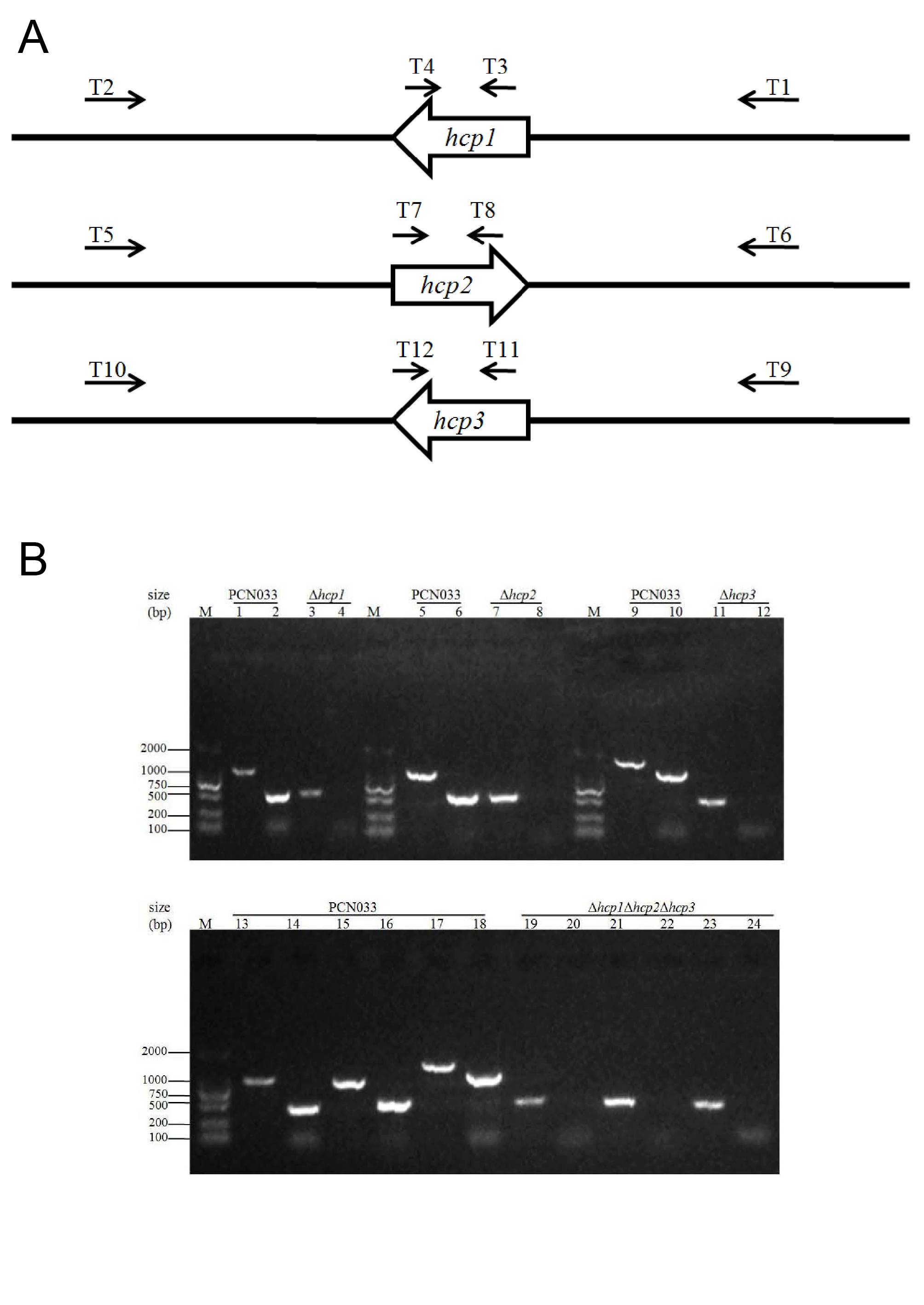


**Supplementary Figure S3.** Dual-enzyme digestion of recombination plasmids for complement strains construction. Recombination plasmids of pHSG::*hcp2* and pHSG::*hcp3* digested by *XhoI* and *KpnI* were displayed in lane 1 and lane 2 successively.


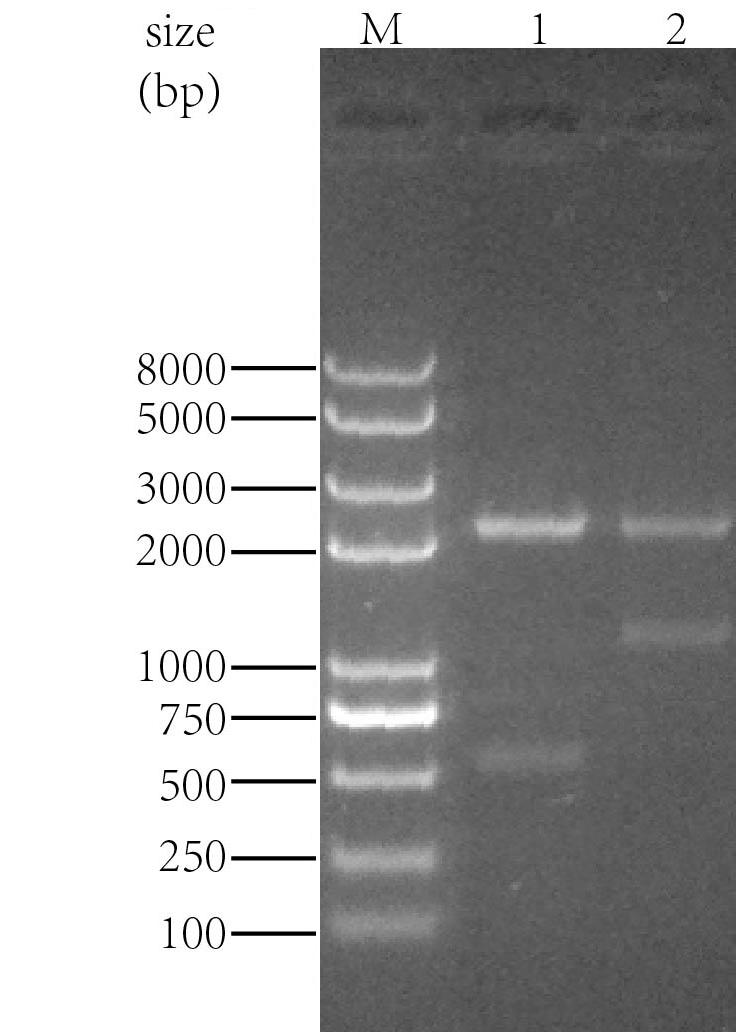


**Supplemantary Table S1.** **Predicted effectors of T6SS in ExPEC strain PCN033 using a tool website** [**http://db-mml.sjtu.edu.cn/SecReT6/**](http://db-mml.sjtu.edu.cn/SecReT6/)**.**

| gene-loci | protein_length | pcn033-ncbi-gene |
| --- | --- | --- |
| complement(246100..246582) | 160 | Query= WP_000002621.1 MULTISPECIES: type VI secretion system protein |
| complement(252932..253675) | 247 | Query= WP_000088866.1 type VI secretion protein [*Escherichia coli*] |
| complement(1793408..1794436) | 342 | Query= WP_000179515.1 LacI family transcriptional regulator [*Escherichia coli*] |
| complement(1377721..1379181) | 486 | Query= WP_000214516.1 MULTISPECIES: cardiolipin synthase[Enterobacteriaceae] |
| 4755158..4756114 | 318 | Query= WP_000265935.1 MULTISPECIES: ABC transporter substrate-binding protein [Enterobacteriaceae] |
| complement(2354502..2355500) | 332 | Query= WP_001036964.1 MULTISPECIES: D-galactose-binding periplasmic protein [Enterobacteriaceae] |
| 397775..398758 | 327 | Query= WP_001042105.1 MULTISPECIES: LacI family transcriptional regulator [Enterobacteriaceae] |
| 4178578..4179468 | 296 | Query= WP_001056269.1 D-ribose transporter subunit RbsB [*Escherichia coli*] |
| 4365258..4365893 | 211 | Query= WP_001094920.1 baseplate assembly protein [*Escherichia coli*] |
| complement(2797951..2798934) | 327 | Query= WP_001124892.1 sugar ABC transporter substrate-binding protein [*Escherichia coli*] |
| 267403..267921 | 172 | Query= WP_001142958.1 MULTISPECIES: hypothetical protein [Enterobacteriaceae] |
| complement(4258749..4259897) | 382 | Query= WP_001280045.1 MULTISPECIES: type VI secretion protein [Enterobacteriaceae] |
| complement(4797423..4798421) | 332 | Query= WP_001309159.1 MULTISPECIES: transcriptional regulator [Enterobacteriaceae] |
| 3937639..3938631 | 330 | Query= WP_001366657.1 MULTISPECIES: xylose ABC transporter substrate-binding protein [Enterobacteriaceae] |
| 4180536..4181519 | 327 | Query= WP_001376108.1 MULTISPECIES: LacI family transcriptional regulator [Enterobacteriaceae] |
| complement(2969663..2970673) | 336 | Query= WP_024210272.1 transcriptional regulator [*Escherichia coli*] |
| 270305..274567 | 1420 | Query= WP_047938332.1 hypothetical protein [*Escherichia coli*] |
| 774741..778916 | 1391 | Query= WP_047938335.1 RHS element protein [*Escherichia coli*] |
| 1615760..1619914 | 1384 | Query= WP_047938343.1 RHS element protein [*Escherichia coli*] |
| complement(2169467..2173648) | 1393 | Query= WP_047938391.1 RHS element protein [*Escherichia coli*] |
